# Predictors and brain connectivity changes associated with arm motor function improvement from intensive practice in chronic stroke

**DOI:** 10.12688/f1000research.8603.2

**Published:** 2017-02-28

**Authors:** George F. Wittenberg, Lorie G. Richards, Lauren M. Jones-Lush, Steven R. Roys, Rao P. Gullapalli, Suzy Yang, Peter D. Guarino, Albert C. Lo

**Affiliations:** 1 Department of Veterans Affairs (VA) Maryland Health Care System, Geriatrics Research, Education and Clinical Center, and Maryland Exercise & Robotics Center of Excellence, Baltimore, MD, 21201, USA; 2 Departments of Neurology, Physical Therapy and Rehabilitation Science, Internal Medicine, Older Americans Independence Center, University of Maryland, Baltimore, MD, 21201, USA; 3 North Florida/South Georgia Veterans Health System, Gainesville, FL, 32611, USA; 4 University of Florida, Gainesville, FL, 32608, USA; 5 Department of Physical Therapy and Rehabilitation Science, University of Maryland, Baltimore, MD, 21201, USA; 6 Department of Radiology, University of Maryland, Baltimore, MD, 21201, USA; 7 VA Cooperative Studies Program Coordinating Center, West Haven, CT, 06516, USA; 8 Providence VA Medical Center and VA Research and Development Center of Excellence, Center for Restorative and Regenerative Medicine, Brown University, Providence, RI, 02908, USA

**Keywords:** Predictors, brain connectivity, robotic, motor function

## Abstract

**Background and Purpose:** The brain changes that underlie therapy-induced improvement in motor function after stroke remain obscure. This study sought to demonstrate the feasibility and utility of measuring motor system physiology in a clinical trial of intensive upper extremity rehabilitation in chronic stroke-related hemiparesis. **Methods: ** This was a substudy of two multi-center clinical trials of intensive robotic and intensive conventional therapy arm therapy in chronic, significantly hemiparetic, stroke patients. Transcranial magnetic stimulation was used to measure motor cortical output to the biceps and extensor digitorum communus muscles. Magnetic resonance imaging (MRI) was used to determine the cortical anatomy, as well as to measure fractional anisotropy, and blood oxygenation (BOLD) during an eyes-closed rest state. Region-of-interest time-series correlation analysis was performed on the BOLD signal to determine interregional connectivity. Functional status was measured with the upper extremity Fugl-Meyer and Wolf Motor Function Test. **Results:** Motor evoked potential (MEP) presence was associated with better functional outcomes, but the effect was not significant when considering baseline impairment. Affected side internal capsule fractional anisotropy was associated with better function at baseline. Affected side primary motor cortex (M1) activity became more correlated with other frontal motor regions after treatment. Resting state connectivity between affected hemisphere M1 and dorsal premotor area (PMAd) predicted recovery. **Conclusions:** Presence of motor evoked potentials in the affected motor cortex and its functional connectivity with PMAd may be useful in predicting recovery. Functional connectivity in the motor network shows a trends towards increasing after intensive robotic or non-robotic arm therapy. Clinical Trial Registration URL: http://www.clinicaltrials.gov. Unique identifiers: NCT00372411 \& NCT00333983.

## Introduction

The development of new methods for rehabilitation of deficits after stroke has enabled research into the brain mechanisms of improved function after such therapy. This has been accomplished in Constraint Induced Therapy ^1, 2^, Bilateral Arm Training ^3^ and in one form of robotic hand training ^4^. Some common themes in these studies include: 1. Changes in motor task-related brain activation after therapy (although both positive and negative changes have been reported) and, 2. Expansion of shrunken motor maps as measured by transcranial magnetic stimulation (TMS) ^5, 6^. However, there remain ambiguities and even controversies regarding the effects of repetitive task practice on brain activity and whether modern, well-defined therapeutic methods differ in their brain effects.

Robotic rehabilitation has certain mechanistic advantages over other therapeutic methods ^7^. Robotic therapy is a better option for more severely affected stroke patients who may not be able to practice certain movements without external assistance. In such patients the mechanisms of recovery may be qualitatively different from those who can independently practice a full range of movements. This group may have the most to gain with improved understanding of the mechanisms of action of repetitive practice. In addition, although robotic therapy is well defined by the algorithms it uses for training, the therapy is flexible enough to train patients in various types of movements.

We had the opportunity to perform a multi-center investigation of the brain mechanisms underlying robotic rehabilitation by studying a subset of participants in two multi-center VA (U.S. Dept. of Veterans Affairs) studies that compared robotic rehabilitation to both an intensity-matched non-robotic therapy regimen and usual care. The hypotheses for this study related to both prognosis (e.g. greater cortical motor excitability and reduction in transcallosal inhibition will predict greater functional improvement) and treatment effects (e.g. intensive rehabilitation will more effectively increase the ability to activate multiple muscles through motor cortical activity, partly through reduced interhemispheric inhibition to the affected motor cortex, and resulting in a shift of the recruitment curve upward and leftward). It was also an opportunity to test a connectivity-based approach that has shown promise in studies of recovery of function ^8, 9^ with the hypothesis that intensive practice would reverse deficits in motor system connectivity.

## Methods

### Clinical trial

**Figure 1.**
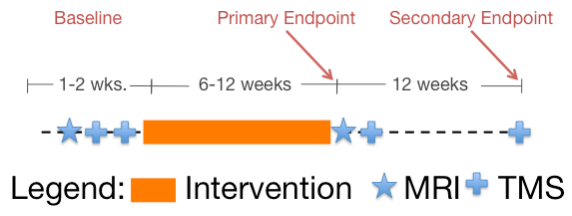
Study Design. The timeline of baseline measures and the therapy interventions are shown graphically. MRI and TMS measurements were performed before or after the intervention.

This was a substudy of two multi-center clinical trials whose methods and results have been published ^10– 12^. Briefly, all participants received intensive task practice, one hour a day, three times a week, for 6 or 12 weeks. The robotic rehabilitation group used a suite of robots (InMotion, Bionik, Watertown, MA) with close to 1000 repetitions performed in a session. The intensive conventional group used non-robotic devices and therapist assisted exercises, often using the less-affected hand or therapist guidance for assistance, with an estimated number of movement cycles above 600 per session. There was also a usual care control group that received therapy, if prescribed already. It was originally intended to enroll approximately 40 participants across four sites but due to regulatory and staffing at issues at two sites, 13 subjects across two sites were enrolled, with no control group subjects enrolled. All participants were chronic hemiparetic stroke patients with a significant degree of impairment (Upper Extremity Fugl-Meyer scale 7–38.) Figure 1 shows the basic design of the substudy, with TMS and magnetic resonance imaging (MRI) measures bracketing a 6–12 week intervention. Clinical Trial Registration URL: http://www.clinicaltrials.gov. Unique identifiers: NCT00372411 & NCT00333983.

### Transcranial magnetic stimulation (TMS)

Stimulation of the motor cortex responsible for upper-extremity impairment was performed using a MagStim 200 or 2002 Magnetic Stimulator (MagStim Ltd., Wales, UK and 70 mm D double circular coil. Motor evoked potentials (MEP) were recorded unilaterally by surface electrodes fixed over the biceps and extensor digitorum communus (EDC) muscles in bipolar montage with 3 cm spacing. Responses were amplified by a battery-powered surface electromiography (EMG) integrated electrode and amplifier (B&L Engineering, Tustin, CA or DelSys, Boston, MA), and fed into a personal computer through a multifunctional I/O board and LabView acquisition/analysis software (National Instruments, Austin, TX). A 100 ms period after stimulus was examined with time window adjusted to capture only the MEP. Amplitudes were measured peak-to-peak. Bandpass was 30–1000 Hz and digitization 2000 Hz.

The target muscle was ensured to be at rest during the entire procedure, through audio and visual monitoring of muscle activity. Motor threshold was determined using International Federation of Clinical Neurophysiology criteria ^13^ except that a 25 *µ*V limit was used because of the bipolar montage. The coil was localized on the frontoparietal region contralateral to the target muscle in the examined limb and moved until each muscle’s hot-spot, where the response threshold was the lowest, was found. Exact position of stimulation was recorded using a stereotactic system (BrainSight, Rogue Research, Montreal, QC, Canada) and guided by a 1 cm Cartesian coordinate system projected onto the subject’s own MRI. If a different hotspot was found on a subsequent visit, threshold and recruitment curves were obtained at both the original and new hotspot, but the original hotspot data were used for group analysis (hotspot location did not vary more than 1 cm in any case).

Recruitment curves were measured by stimulating at a range of intensities, from 10% below threshold, increasing in increments of 10% of threshold until the response plateaued or the maximum output of the stimulator was reached. Ten stimuli at each intensity were delivered.

### Ipsilateral silent period

The ipsilateral silent period ^14^ was measured by stimulation of the unaffected (defined here as contralesional) cortex and voluntary activation of the affected arm. Maximum weight that the subject could sustain in each target muscle was determined. For the biceps the weight was placed on the wrist and for the extensor digitorum communis (EDC) on the proximal interphalangial joints. The coil was placed over the hand knob (hand representation within M1) of the unaffected hemisphere. After the subject stabilized the weight against gravity, a TMS pulse 100% of the maximal stimulator output was delivered. The subject was allowed to rest for several seconds and the procedure was repeated two further times. The EMG signal was integrated, and a ratio of pos- trelative to pre-stimulation activity was computed, making the appropriate adjustment for the length of period.

### Magnetic resonance imaging (MRI)

Anatomical and Functional Magnetic Resonance Imaging was performed at each center on a Tim Trio 3T scanner (Siemens AG, Erlangen, Germany) equipped with an 8-channel receive-only head coil. Anatomical Imaging: These consisted of a high-resolution three-dimensional sagittal T1-weighted magnetization-prepared rapid gradient echo (MP-RAGE) image, and oblique proton density and T2-weighted images acquired with 2 mm slice thickness. Diffusion-tensor images were acquired using a single-shot echo-planar technique and 65 directions. A b value of 1000 s/mm ^2^ was used with an average of six images acquired to increase signal-to-noise ratio. A fractional anisotropy (FA) map was created from these data. A 5 mm radius spherical Region of Interest (ROI) was centered on the posterior limb of the left and right internal capsules (IC) on the FA images and the mean, standard deviation, and ratio (affected/unaffected) were computed. While a spherical ROI is not ideal for the IC, this was a limitation of the analysis method, but also enforced objectivity in contrast to a customized ROI. One author (GW) drew all of the ROI, and all analysis was performed in the Baltimore imaging center.

**Functional Imaging.** Two eyes-closed rest scans were obtained, each with 128 coronal blood oxygenation-level dependent (BOLD) weighted volumes (echo planar imaging; 3 sec TR, 30 ms TE, 4 mm slice thickness with no gap, flip angle = 90°, 36 axial slices, 1.8 × 1.8 mm ^2^ inplane resolution, FOV = 23 cm). These were separated in time by at least 5 minutes. A tape and cushion technique was employed to reduce head motion and remind the subjects of the need to keep their head as still as possible. We examined head motion parameters within the analysis and rejected runs with absolute head movement greater than 2 voxels. Images were corrected for head motion by realignment and Independent Component Analysis (ICA) was used to remove movement related signal ^15^.

**Figure 2.**
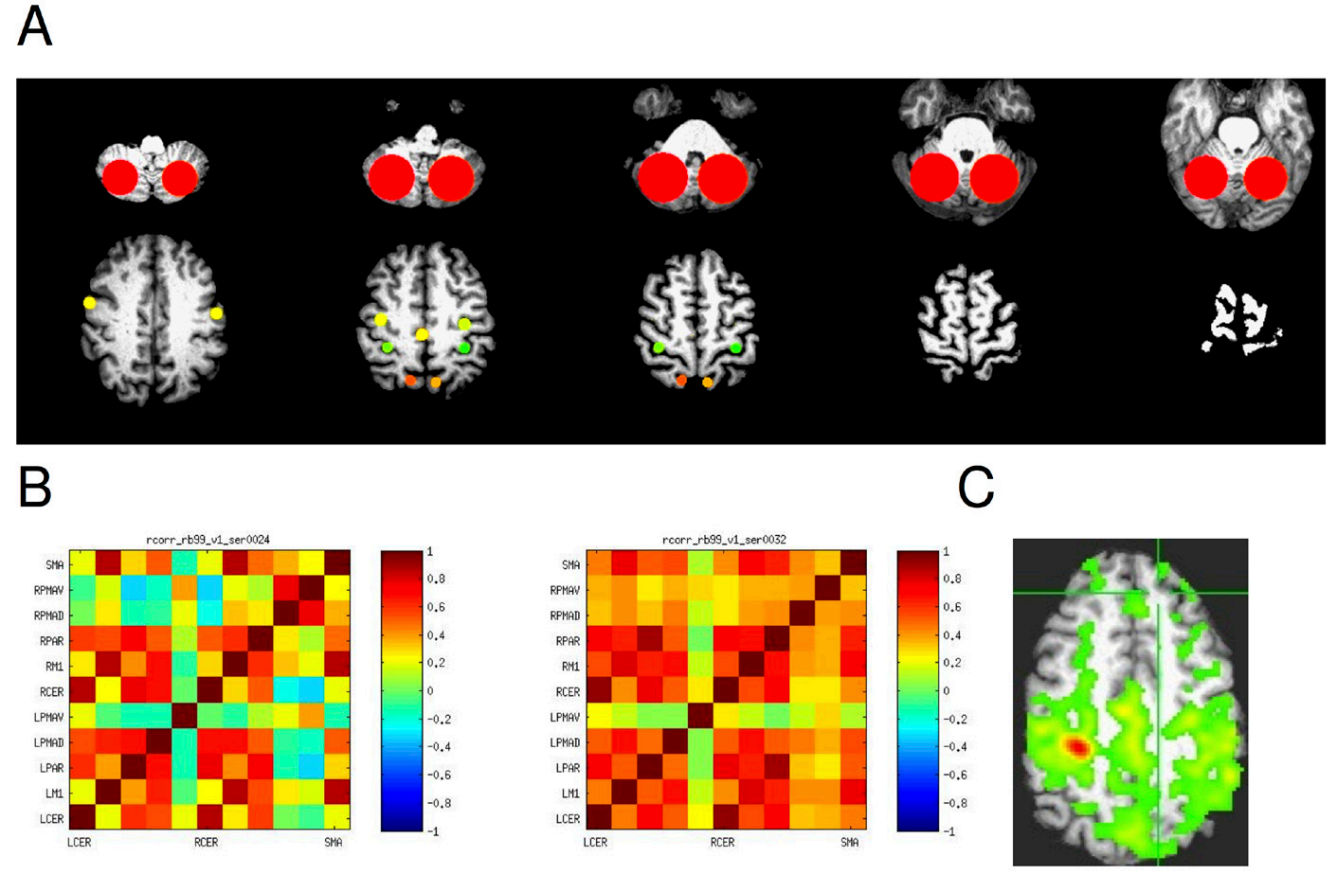
Resting state connectivity (correlations.) **A**: The 11 regions of interest (ROIs) are shown on representative axial slices of an example brain MRI. The top slices show the cerebellar ROI, then the PMAv on the left bottom slice, and PMAd, M1, and superior parietal regions from anterior to posterior. The SMA is represented by a single midline ROI. **B**. Correlation matrix with correlations at baseline in two resting states scans in the same participant. **C**. Example correlation in a single slice with a affected side M1 ROI as the seed.

**Region of Interest (ROI) resting state correlation analysis.** Normalization of individual brains to an atlas template always introduces inaccuracies, but normalization of brains with lesions present particular challenges that require compromises or lesion masking. Therefore, an ROI based analysis was performed without spatial normalization in AFNI ^16^ and MATLAB (MathWorks Inc., Natick, MA). All of a participant’s resting state scans were corrected for slice timing and spatially registered to the first resting sate scan from their first session. The structural image was skull-stripped and also spatially registered to the subject’s first functional scan. A 6-mm FWHM Gaussian blur was then applied to all spatially registered EPI scans. Nine ROIs were selected manually identifying the following anatomical landmarks: medial part of the precentral gyrus, postcentral gyrus, cerebellar hemispheres, supramarginal gyrus, supplementary motor area (caudal supplementary motor area between medial precentral gyrus and a coronal plane through the anterior commisure ^17^ and superior, middle, and inferior frontal gyri, Figure 2. Pairwise ROI correlations were computed on the time series for each ROI and Z-transformed.

### Statistical analysis

SAS (Cary, North Carolina) was used for all analysis. Pearson correlation was used to calculate recruitment curve slope and associations between variables. Mixed models (REML with compound symmetry) were used to analyze predictive factors such as presence of TMS responses and recruitment curve slope.

## Results

### Functional outcome and TMS

**Figure 3.**
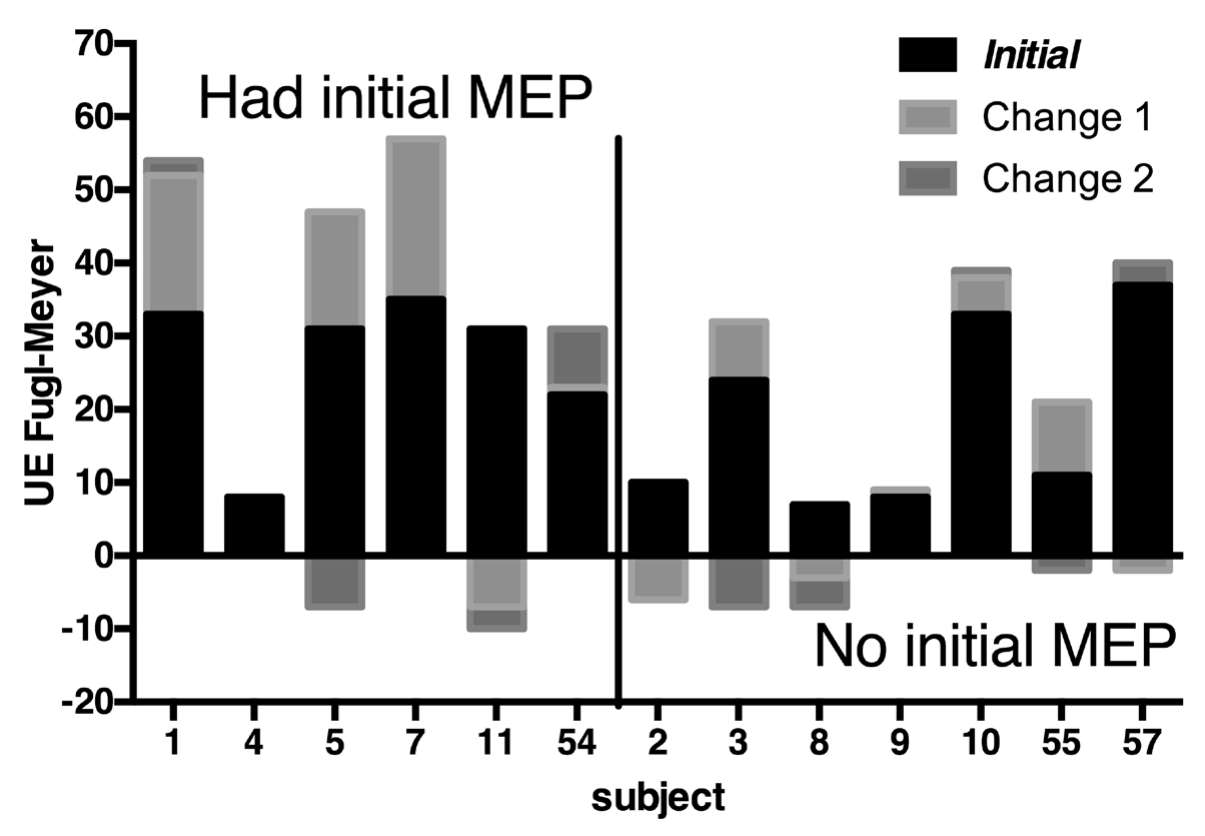
Clinical Outcomes. The baseline and change in Fugl-Meyer score are shown for each participant, grouped by whether there was initial motor evoked potential as measured by TMS. Change 1 is across the intervention; Change 2 is between the end of the intervention and the last outcome measurement (12 weeks.) Negative changes are always shown below the baseline. Source data is in Dataset1.

Fourteen subjects were enrolled between 2008 and 2010 at the Baltimore and North Florida/South Georgia VAMC. Thirteen were stroke patients, three of whom were randomized to intensive comparison therapy and ten of which were randomized to robot therapy, and one subject was a healthy control who received no therapy. The relationship between initial and follow-up Fugl-Meyer (FM) impairment score and presence of MEP is shown in Figure 3. While MEP were absent in most of the lower functioning participants initially, there were both low and high functioning participants with absent and present MEP. Controlling for the effects of baseline FM in a fixed effects analysis, presence of an MEP at baseline was associated with a mean 3.3 *±* 6.2 S.E. (N.S.) higher change in FM across all post-baseline visits. (There were up to four post-baseline visits.) A biceps MEP was never present without an EDC MEP, but not *vice versa* and the predictive value of these two measures was approximately equal.

### TMS: recruitment curves

**Figure 4.**
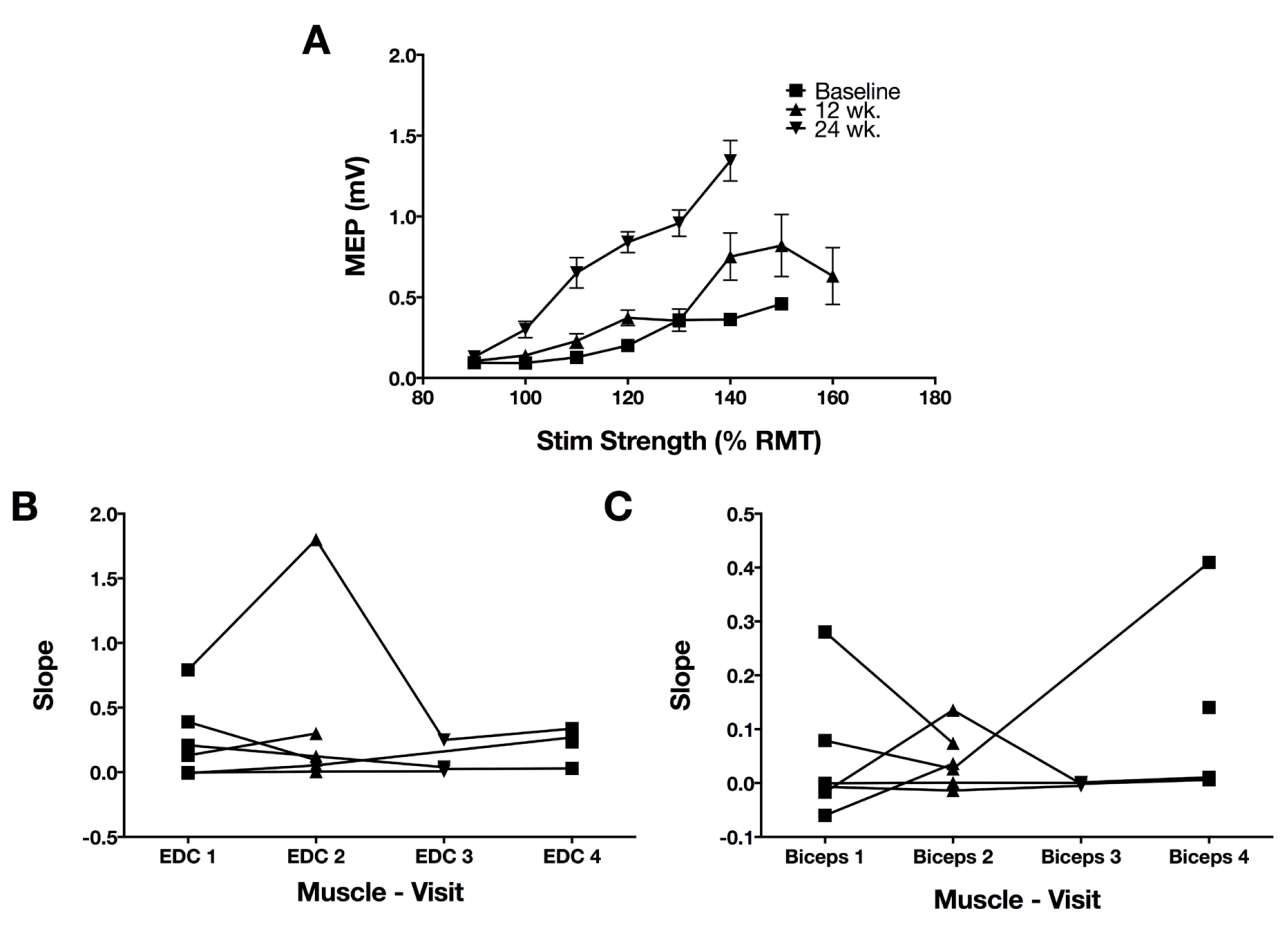
Recruitment curves on the affected upper extremity. **A**. Single participant (#1) recruitment curves in the EDC and the second baseline and two follow-up measurements. Stimulation strength is indicated on the x-axis as a percentage of resting motor threshold. Raw data is in Dataset2-ExampleRecCurve|. **B**. The slope of the recruitment curve between 100 and 120% of resting motor threshold stimulation strength was extracted from each recruitment curve of each participant that had measurable recruitment curves that could be measured on the affected side EDC. The first two measurements are both baseline periods. Changes in the recruitment curve slope were not significant. **C**. Recruitment curve slope in biceps (otherwise, as in **B**.) Data is in Dataset2-RecCurvSlope.

One of the hypotheses regarding recruitment curves was that steeper recruitment curves would correlate with better function. However, there were no significant correlations between either EDC or biceps recruitment curves and function, within the population in which recruitment curves could be evaluated (N=6.) Almost all participants had very shallow recruitment curves, with one moderately affected individual (FM=35) being the exception ( Figure 4). There was a non-significant trend for higher recruitment curve slope at baseline correlating with functional improvement in a mixed effects model that controlled for baseline FM.

### Silent periods

**Figure 5.**
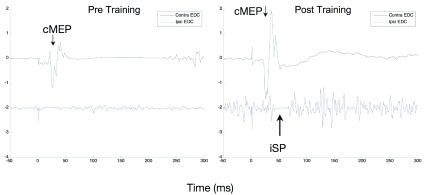
Ipsilateral Silent Period. An example of ipsilateral silent period measured at baseline (left) and immediately after the 12 weeks intervention (right) in participant RB5. EMG is measured in mV in the EDC muscle contralateral to the TMS stimulator in the upper trace and ipsilateral in the lower trace, which is offset by 2 mV. Before the intervention there is little activation of the muscle and also no apparent silent period. There is much more activation of the muscle after the intervention and also a visible short ipsilateral silent period.

A long-lasting stimulus artifact contaminated too many cases to allow group analysis. One example of change in silent period is noted in Figure 5. In this case the subject had an increase in voluntary activity after the intervention, despite the same amount of force requirement, and demonstrated a clearer iSP only after the intervention. However, besides in two other subjects, there was no visible iSP.

### RSC analysis

**Figure 6.**
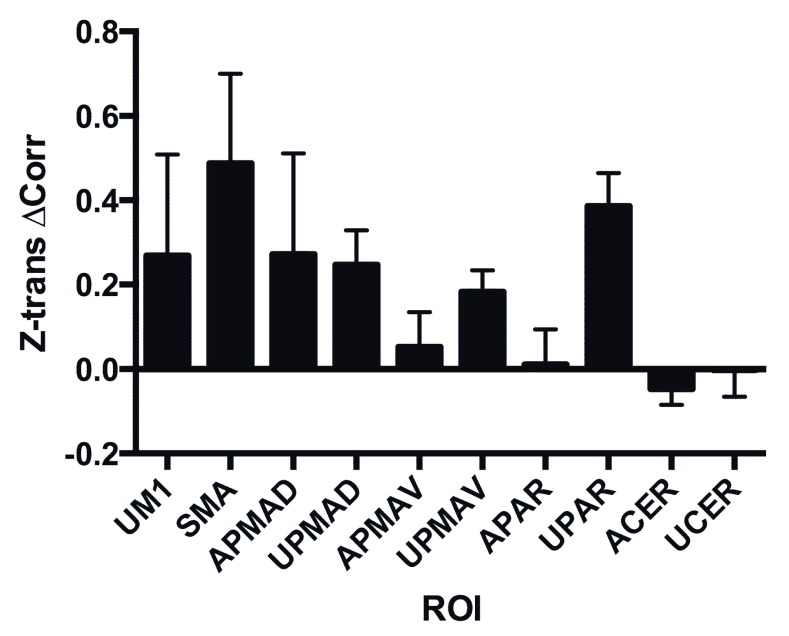
Change in connectivity of AM1. The mean change and S.D. of the Z-transformed correlation coefficient of the affected primary motor cortex (AM1) with each of the other regions is shown. Note that all regions showed an increase in connectivity except the parietal regions. Region names include ‘A’ for affected side hemisphere (the side opposite to the affected hemisphere in the case of the cerebellum, ‘CER’) and ‘U’ for unaffected, with region names as in Figure 2.

***Predictive measures.*** Resting state analysis resulted in a correlation matrix for the chosen ROI. While there was not an age-matched control population, there were clear asymmetries in the correlation matrices, as within hemisphere connections were decreased on the affected side as compared to the unaffected, but with exceptions such as the parietal area (data not shown). We were particularly interested in exploring the changes of correlation of the affected side motor cortex (M1) with other brain areas. Correlation of the affected M1 with all frontal lobe motor regions increased over the course of treatment, but there was no change in correlation of M1 with the cerebellum ( Figure 6). The change in the unaffected M1, SMA and the unaffected side superior parietal area were most striking.

***Correlative measures.*** Improvement in Fugl-Meyer correlated with a trend towards reduction in two pairwise correlations. Greater FM increase was associated with decrease in affected M1-unaffected M1 connectivity ( *r* ^2^=0.31, p = 0.07), and unaffected M1-affected superior parietal area ( *r* ^2^=0.34, p = 0.06). All other changes in functional connectivity measures correlated less well with changes in connectivity.

***MRI: Fractional anistropy of the corticospinal tract.*** FA of the affected internal capsule was correlated with baseline motor ability ( *r* ^2^ = 0.48, p < 0.01.) but did not predict motor recovery, although the trend was for greater FA to be associated with better recovery.

There were no significant differences in any outcome measure for robotic vs non-robotic comparison therapy ( Dataset 1 for Fugl Meyer, other outcomes not shown).

Raw data for predictors and brain connectivity changes associated with arm and motor function improvement from intensive robotic practice in cronic strocke Descriptions of each dataset are provided in the readme file. ^28^. Click here for additional data file. Copyright: © 2017 Wittenberg GF et al. 2017 Data associated with the article are available under the terms of the Creative Commons Zero "No rights reserved" data waiver (CC0 1.0 Public domain dedication).

## Discussion

The purpose of this study was to obtain feasibility data on the use of TMS and MRI to provide predictive and mechanistic information about the motor functional response to intensive arm rehabilitation. It was not expected to provide definitive results in a field that has been marked by inconsistency. Some of the lessons learned in this study are: 1. The lack of TMS responses in a majority of the moderate-to-severe population limits the utility of TMS for measuring change, although when MEP are present this predicts a better response to intervention, as has been demonstrated previously ^18^; 2. The ipsilateral silent period, a measure of transcallosal inhibition that can be performed even when MEP cannot be elicited, has limitations as well, and was not useful in this particular study, partly for technical reasons. 3. MRI measures of resting state connectivity were more revealing, demonstrating both the deficits and changes with therapy, although in a purely exploratory manner. Again, these conclusions are limited by the small sample size, and by a delay in adding TMS and MRI measures to a clinical trial that closed a control group to recruitment before these measures were added.

The recruitment curve is a standard metric of effectiveness of the corticospinal tract, and could have been useful in this study in demonstrating a mechanism of practice-related plasticity. There are a few possible reasons why it didn’t: 1. The training may have had no consistent effect on recruitment curves or there are diverse mechanisms of reduced motor impairment; 2. The lack of MEP in many participants made the recruitment curve immeasurable, further reducing the power of the analysis; 3. the very flat recruitment curves measured in some participants reflect a reduction in direct cortical control of lower motor neurons, with the reticulospinal system having more influence, and that system not being activated well by M1 TMS after stroke.

### Silent period and recovery of cortical control

There are two types of silent period measurement: intracortical and ipsilateral. The former is performed with the coil over the motor cortex that is preactivating the muscle, and the lateral over the opposite hemisphere (so ipsilateral to the muscle being preactivated). The silent period is a very convenient measure of intracortical and interhemispheric inhibition because only a single pulse from a single coil is required. It is a complicated measure to interpret because it depends in the level of voluntary activation, and as with almost all TMS measures, is dependent on surface EMG recordings. There are also different findings for ipsilateral silent period depending on the muscle tested ^19^. Stroke can affect both the level of cortical activation and muscle activity. Still, the intracortical silent period has been a useful measure in stroke ^20^ and the ipsilateral silent period as well ^21^.

While there were technical limitations to use of silent periods, as shown in Figure 5, the silent period could become more apparent after therapy. Since the appearance of a silent period depends on cortical activation of an affected muscle, a silent period could appear to be absent if there is little such cortical activation. Increased corticomotor effectiveness can thus result in the appearance of a silent period, and give a misleading impression that more intercortical inhibition is related to better function. The role of intercortical inhibition in shaping motor function is complex, and interpretation of tests that require activity in interhemispheric networks needs to be sophisticated.

### Role of superior parietal cortex in recovery and compensation

In the resting state connectivity analysis, all connectivity with the affected motor cortex was negatively correlated with impairment. The two exceptions were the superior parietal area, in which increased connectivity was correlated with impairment. There have been a number of reports of the role of the superior parietal area in recovery of function after stroke ^22– 26^. However, its correlation here would suggest an association with more impairment and a role in compensation in only the more severely affected individuals.

### Changes in connectivity

The correlation matrix for even a small number of ROIs is a large amount of data and causes a multiple comparison problem. We focused on connectivity with the affected motor cortex as being most relevant to recovery of function. Out of the ten other regions, correlation with eight of them increased over the course of therapy. The largest increases were in the contralesional superior parietal area, ipsilesional dorsal premotor area, and supplementary motor area. These regions have strong bilateral connections and are good candidates for brain regions that would be engaged by the practice involving visual motor feedback and proximal arm movements. There were no significant associations between change in interregional correlation and a change in motor function. The best correlation with recovery was in connections of the unaffected M1 with two areas in the affected hemisphere: affected M1 and affected superior parietal area. The fact that this was a negative correlation suggests that intensive unimanual therapy may be decreasing the importance of the unaffected primary motor area in movement of the affected side. But other changes, not measured in this study, may be related to recovery of function, and the measured network changes, whether or not they are a significant effect of the intervention, may not be necessary for recovery or may represent compensatory changes. Likely because of the relatively small size of the study, were not able to find significant differences between such changes in the two types of treatment, if any differences exist. It would be interesting to speculate that the superior parietal activity would be more involved in robotic rehabilitation, with its visuomotor component and SMA in the intensive comparison treatment that involved more self-initiated activity.

**Table 1.** Demographic information of study participants who completed the study. Subject numbers started at 1 in Baltimore, and at 50 in Florida. All had anterior circulation ischemic strokes except for #1 who had a thalamic hemorrhage, which did make #1 an outlier. Therapy assignment included either robotic or intensive comparison (comp.) therapy. When length of therapy was 6 weeks, there was no therapy with the wrist robot, only planar and vertical robots.

Subject	Age	Gender	Arm Aff.	Group	Duration	FM baseline	FM 6 or 12 wk
1	44	M	L	Robot	12	33	53
2	64	M	R	Comp.	12	10	4
3	61	M	L	Comp.	12	24	32
4	54	M	R	Comp.	12	8	8
5	81	M	R	Comp.	12	30	47
7	48	M	L	Robot	12	35	57
8	69	M	R	Robot	12	7	4
9	58	M	L	Comp.	6	8	9
10	51	M	R	Robot	6	33	38
11	52	M	R	Robot	6	31	24
54	45	F	R	Comp.	12	22	23
55	71	M	R	Robot	12	11	21
57	70	M	R	Comp.	12	37	35

## Conclusions

Measurement of brain changes related to motor recovery in moderate-to-severely affected stroke patients is complicated by difficulties in measuring brain function noninvasively. But our study showed that simple MEP presence might be useful in predicting response to rehabilitation in chronic stroke, while resting state connectivity appears to be responsive to treatment, with increase in affected primary motor cortical connectivity to other frontal motor areas. Motor cortical functional connectivity with the superior parietal cortex may be marker for compensatory changes that do not respond to affected side intensive practice.

## Data availability

The data referenced by this article are under copyright with the following copyright statement: Copyright: © 2017 Wittenberg GF et al.

Data associated with the article are available under the terms of the Creative Commons Zero "No rights reserved" data waiver (CC0 1.0 Public domain dedication). 

F1000Research: Dataset 1. Raw data for predictors and brain connectivity changes associated with arm and motor function improvement from intensive robotic practice in chronic stroke, 10.5256/f1000research.8603.d133175 ^28^

## Supplementary material

**Supplementary Figure 1.** Correlation between fractional anisotropy (FA) of the affected hemisphere’s internal capsule ROI and baseline Fugl-Meyer score. Higher FA values were associate with better function at baseline. Linear Regression line and 95% confidence intervals are shown. N =13 (Data included all scans available, so > 13 points are plotted). Deviation from zero for slope of line is significant at p=0.008.
