# Supplementary figures and images for: Predictors and brain connectivity changes associated with arm motor function improvement from intensive practice in chronic stroke
Source: F1000Res. 2017 Feb 28;5:2119. Originally published 2016 Aug 31. [Version 2] doi: 10.12688/f1000research.8603.2 (PMC5345776; doi:10.12688/f1000research.8603.2)

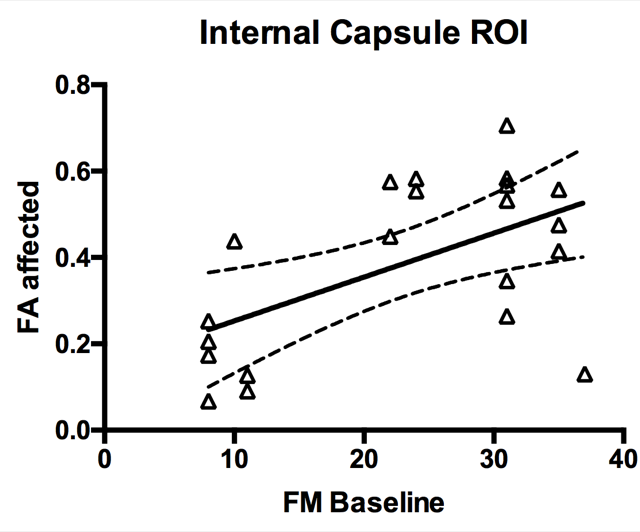

Supplement: Supplementary file 2 [file f1000research-5-11828-s0001.tgz › 3ab92968-1d67-4334-bd9e-1b01552a455a.tiff]
