# Supplementary material for: Predictors and brain connectivity changes associated with arm motor function improvement from intensive practice in chronic stroke
Source: F1000Res. 2017 Feb 28;5:2119. Originally published 2016 Aug 31. [Version 2] doi: 10.12688/f1000research.8603.2 (PMC5345776; doi:10.12688/f1000research.8603.2)
Supplement: Peer review history

# Peer review: Referee response for version 2

**DOI:** 10.5256/f1000research.11828.r20593

**Published:** 2017-03-07

**Authors:** Rudiger Seitz

**Affiliations:** 1 Department of Neurology, Heinrich Heine University Düsseldorf, Düsseldorf, Germany

Careful revision of an interesting paper.

I have read this submission. I believe that I have an appropriate level of expertise to confirm that it is of an acceptable scientific standard.

# Peer review: Referee response for version 1

**DOI:** 10.5256/f1000research.9257.r16609

**Published:** 2016-10-24

**Authors:** Sean Dukelow, Jennifer Semrau

**Affiliations:** 1 Department of Clinical Neurosciences, University of Calgary, Calgary, AB, Canada

The authors present an interesting paper that examines neurophysiologic measurements in 13 chronic stroke survivors who completed robotic therapy or intensive interventions lasting either 6 weeks or 12 weeks. The study explores the use and predictive capabilities of MEPs, FA, and resting state fMRI. Further there is some discussion of the ipsilateral silent period but this was difficult to obtain in a number of subjects for technical reasons. Although we are enthusiastic about the study, we have some concerns over the manuscript in its existing format and put forward a number of questions/suggestions for the authors below.

  

- The authors used a number of different measures – it would have been nice to see a hypothesis associated with each of these measures.

- Could the authors please be more clear on the rehabilitation intervention? We recognize that they do cite the trials from which the data was taken, but even a line or two discussing what went on in the robot vs the comp. groups would be helpful. It would also be helpful to demarcate which individual received what therapy in Figure 3.
- The authors state that the ROI analysis for the resting state fMRI was performed without normalization. Those unfamiliar with this type of analysis may not understand why normalization is not required. Please add a sentence or two to provide justification.
- How often did the authors note a change in the motor hotspot using TMS?
- Why use a 5mm spherical region of interest for the PLIC as the PLIC is not a spherical structure? Some explanation of the reasoning for this would be helpful.
- What was the role of the single control subject who is mentioned in the methods? Please clarify.
- Exactly how many subjects did the authors find/not find an ipsilateral silent period in?
- Re: Fractional Anisotropy (FA): R-squared = 0.48 is reported for the relationship of FA to baseline motor ability. It would be nice to see this in a scatter plot as the relationship is quite strong.
- The manuscript might benefit from some discussion of why steeper MEP recruitment does not lead to better function.
- The authors mention the technical limitations of using silent periods. For readers who are not familiar with this technique, a sentence or two briefly describing what those limitations are would be helpful. Further, the discussion of the importance of the silent period relative to what is known in the literature would be helpful.
- We would like to see slightly more discussion about the limitations of an n=13 sample size. For instance, the results that those subjects with an MEP at baseline tended to do better appears to be driven by 3 subjects based on figure 3.

 

*Minor Concerns:*

- Many abbreviations go undefined in the paper:

1. Last paragraph of introduction: It would be appreciated if the authors provided the references for the two multicenter VA studies that they discuss. They should also define VA for readers.
2. Figure 2 – the authors need to state what the abbreviations are in the text for the correlation matrices.
3. Figure 6 – Please label your abbreviations.

-  Dataset 1 label: Please check your spelling “cronic strocke”  
- In the PDF version of the manuscript, the quality of the correlation matrices in Figure 2B appears to be low resolution.
- Introduction: “In such patients the mechanisms of recovery may be qualitatively different and who have the most to grain with improved understanding of the mechanisms…” – this sentence appears to be incomplete.

We have read this submission. We believe that we have an appropriate level of expertise to confirm that it is of an acceptable scientific standard, however we have significant reservations, as outlined above.

# Response

**Published:** 2017-02-14

**Authors:** George Wittenberg

**Affiliations:** Department of Neurology, University of Maryland, USA

Please see the overall comments to readers. The specific responses to your comments include:

- The hypotheses are stated in the introduction - added the hypothesis about functional connectivity. We elaborated a little more in response to this comment.
- *even a line or two discussing what went on in the robot vs the comp. groups would be helpful* - added.
- Demarcate which individual received what therapy in Figure 3 - still don't want to have comparisons between types of therapy. 9, 54, and 57 and conventional therapy
- Normalization issue explained.
- Addressed hotspot with comment in methods.
- This was a limitation of AFNI (now addressed in paper.
- Spherical ROI explained.
- Addressed numbers of silent periods found.
- FA correlation with impairment can be added as supplemental graph.
- Thanks for the suggestion about recruitment curves. This has been added to the discussion.
- There already was discussion of the requirement for cortical activation, but this has been increased.
- MEP presence wasn't even a significant predictor, just a trend, so it didn't seem worthwhile pointing out. But need add more about limitations.

Minor concerns:

- The references are provided in the first part of the Methods. VA is now defined.
- Abbreviations in Fig. 2 are explained.
- Fig. 6 abbrevs labelled.
- "Cronic strocke" was a filename, but can correct if needed.
- The source data for the correlation matrices had that resolution but it didn't limit information.
- Thanks for noting the run-on sentence. This error was carried forward over many versions but was an awkward construction/splicing job that has now been fixed by splitting the sentence.

# Peer review: Referee response for version 1

**DOI:** 10.5256/f1000research.9257.r15975

**Published:** 2016-09-19

**Authors:** Rudiger Seitz

**Affiliations:** 1 Department of Neurology, Heinrich Heine University Düsseldorf, Düsseldorf, Germany

The authors present a well-designed, multimodal study on the effect of robotic practice in 13 chronic stroke patients using transcranial magnetic stimulation (TMS), high resolution magnetic resonance imaging (MRI), diffusion tensor imaging and resting state BOLD imaging. Investigated variables were motor evoked potentials (MEPs) on the affected side, silent period after TMS of the contralesional motor cortex, fractional anisotropy (FA) of the ipsilesional internal capsule, and functional connectivity of motor cortex in the affected hemisphere using anatomically based regions of interest (ROIs). The main results were that MEPs were associated with better functional outcome, FA of internal capsule was associated with better function at baseline, and BOLD in motor cortex was more correlated with other motor areas after training of which resting connectivity between motor cortex and dorsal premotor cortex predicted recovery. There are some issues that need clarification.

  

- Table 1 shows that 6 patients received robot training, while 7 received intensive comparison therapy. At no other instance it is said what intensive comparison treatment is. In fact, the entire manuscript including the title argues for robot training and presents the data as if all patients received robot training. It is not stated if the two treatments resulted in the same or different motor function. Further, a formal comparison of the two groups also concerning the studied variables is lacking.
- Moreover, patient 1 differed from the other patients as he was the only one with an intracerebral hemorrhage and a subcortical location of the lesion. It should be added how many of the other patients also had subcortical and cortical infarct lesions, respectively.
- Were the imaging data analyzed in one centre or in the participating centres? Were the ROIs drawn by one of the authors or by different authors? What was the interobserver reliability?
- How many MRI slices did the ROIs listed in the methods include?
- What is meant with "predictive values of the MEP measures were approximately equal (page 5)"? Please, be specific and provide the data.
- The increase of connectivity with the superior parietal area is noteworthy, since this is a brain area with profound somatosensory function. The authors should provide information about the sensory deficits of their patients.
- Figure 4 should provide information about which patients are presented in parts B and C.

I have read this submission. I believe that I have an appropriate level of expertise to confirm that it is of an acceptable scientific standard, however I have significant reservations, as outlined above.

# Response

**Published:** 2016-09-21

**Authors:** George Wittenberg

**Affiliations:** Department of Neurology, University of Maryland, USA

Thank you for the careful reading of this manuscript. We should be able to respond to all of the comments directly in the manuscript. But to immediately answer two of the comments:

- The intensive comparison therapy was described in other papers, and we will make that more clear and add a summary. We in no way wanted to give the impression that any changes were specifically related to the type of intensive therapy. That was a possibility, of course, but we did not find that to be true, and had too small a sample to have the power to do so unless it was a truly dramatic difference.
- All MRI analysis was done at one center and ROI drawn by the first author. It was a simple, consistent approach to the data.

# Response

**Published:** 2017-02-14

**Authors:** George Wittenberg

**Affiliations:** Department of Neurology, University of Maryland, USA

Please see the overall comments to readers. The specific responses to your comments include:

- Because of the size of the two groups and the lack of significance of difference between the two active treatments, the data was pooled. While we did take look to see if there were intergroup differences, as expected, this was too small a sample. But we have clarified that all participants received "intensive repetitive task practice" rather than robot therapy.
- Note about outlier was made. Unfortunately is has been hard to track down all of the data regarding stroke location.
- All ROI drawn in one centre with ROI by one author.
- Slice numbers per ROI can be calculated and so was not added to the paper: Since slice thickness was 4 mm for functional imaging and 2 mm for anatomical and DTI imaging, the 5 mm  radius included 5 slices for anatomical scans and 3 slices for functional scans. The cerebellar ROI was four times larger, and had so had 10 slices for the functional image.
- "What is meant with "predictive values of the MEP measures were approximately equal (page 5)"? Please, be specific and provide the data." – I don't see this in the manuscript - maybe in an earlier version?
- Unfortunately we don't have detailed sensory information.
- In Figure 4, Participant #1 is reference and the other data is mean.

# Peer review: Referee response for version 1

**DOI:** 10.5256/f1000research.9257.r15971

**Published:** 2016-09-12

**Authors:** Argye E. Hillis

**Affiliations:** 1 John Hopkins School of Medicine, Baltimore, MD, USA

This is a well-written and informative substudy of a thoughtfully designed clinical trial to improve motor function. The authors report imaging predictors of recovery before and after controlling for initial severity. 

 Main criticism:

- It was not clear that resting state sequences masked infarct. How many patients had infarct in M1. It would be unsurprising that there would be lower connectivity between M1 on the affected side and other areas if it is partially infarcted, and that would predict recovery.
- Conclusions about M1 connectivity predicting recovery are too strong, since the results were not statistically significant, but only showed a trend (e.g. p = 0.06). Furthermore, it seems that they did not control for multiple comparisons, so these could have been found just by chance.

I have read this submission. I believe that I have an appropriate level of expertise to confirm that it is of an acceptable scientific standard, however I have significant reservations, as outlined above.

# Response

**Published:** 2016-09-13

**Authors:** George Wittenberg

**Affiliations:** Department of Neurology, University of Maryland, USA

The resting state analysis was a very simple ROI based one. The ROI were registered by hand to M1 and if it was infarcted, the ROI included the infarct. In the the group, the strokes were predominantly subcortical and the analysis measured changes in connectivity, so this method was appropriate.

 The comment about the limitations are well-taken. We also introduced confusion on many levels by labeling two paragraphs "predictive" and "correlative". The predictive section presents data on longitudinal changes but doesn't state statistics, although it shows S.E in the graph. It also perpetuates a common misuse of the term "predictive." In fact, we did not find RSC data that predicted response to the intervention, although that was a goal of the study. This can be corrected in a version after other reviews.
